# Knee Joint Menisci Are Shock Absorbers: A Biomechanical *In-Vitro* Study on Porcine Stifle Joints

**DOI:** 10.3389/fbioe.2022.837554

**Published:** 2022-03-17

**Authors:** Andreas M. Seitz, Jonas Schwer, Luisa de Roy, Daniela Warnecke, Anita Ignatius, Lutz Dürselen

**Affiliations:** Institute of Orthopedic Research and Biomechanics, Center of Trauma Research Ulm, Ulm University Medical Center, Ulm, Germany

**Keywords:** knee, joint, meniscus, shock absorber, shock, impact, dynamic mechanic analysis (DMA), *in vitro*

## Abstract

The aim of this biomechanical *in vitro* study was to answer the question whether the meniscus acts as a shock absorber in the knee joint or not. The soft tissue of fourteen porcine knee joints was removed, leaving the capsuloligamentous structures intact. The joints were mounted in 45° neutral knee flexion in a previously validated droptower setup. Six joints were exposed to an impact load of 3.54 J, and the resultant loss factor (*η*) was calculated. Then, the setup was modified to allow sinusoidal loading under dynamic mechanical analysis (DMA) conditions. The remaining eight knee joints were exposed to 10 frequencies ranging from 0.1 to 5 Hz at a static load of 1210 N and a superimposed sinusoidal load of 910 N (2.12 times body weight). Forces (F) and deformation (l) were continuously recorded, and the loss factor (tan δ) was calculated. For both experiments, four meniscus states (intact, medial posterior root avulsion, medial meniscectomy, and total lateral and medial meniscectomy) were investigated. During the droptower experiments, the intact state indicated a loss factor of *η* = 0.1. Except for the root avulsion state (−15%, *p* = 0.12), the loss factor decreased (*p* < 0.046) up to 68% for the total meniscectomy state (*p* = 0.028) when compared to the intact state. Sinusoidal DMA testing revealed that knees with an intact meniscus had the highest loss factors, ranging from 0.10 to 0.15. Any surgical manipulation lowered the damping ability: Medial meniscectomy resulted in a reduction of 24%, while the resection of both menisci lowered tan δ by 18% compared to the intact state. This biomechanical *in vitro* study indicates that the shock-absorbing ability of a knee joint is lower when meniscal tissue is resected. In other words, the meniscus contributes to the shock absorption of the knee joint not only during impact loads, but also during sinusoidal loads. The findings may have an impact on the rehabilitation of young, meniscectomized patients who want to return to sports. Consequently, such patients are exposed to critical loads on the articular cartilage, especially when performing sports with recurring impact loads transmitted through the knee joint surfaces.

## 1 Introduction

Patients suffering from anterior cruciate ligament (ACL) injuries (Gillquist and Messner, 1999; Lohmander et al., 2007; Bodkin et al., 2020), meniscus tears (Roos et al., 1998; Roos et al., 1999; Lohmander et al., 2007), or articular fractures of the knee (Weigel and Marsh, 2002; Buckwalter and Brown, 2004; Buckwalter et al., 2004) are up to 20 times more likely to develop post-traumatic osteoarthritis (PTOA) (Muthuri et al., 2011; Carbone and Rodeo, 2017; Thomas et al., 2017) compared to the healthy population. Traumatic meniscal tears are a frequent cause of disability and loss of work time (Mccann et al., 2009; Mcdermott, 2011; Badlani et al., 2013; Carbone and Rodeo, 2017), mostly affecting patients under 40 years of age (Ridley et al., 2017) and professional athletes (Majewski et al., 2006; Yeh et al., 2012; Snoeker et al., 2013). One of the superior goals of this active patient group is to return to work or sports as soon as possible. While arthroscopic suture repair provides a good prognosis for tears in the blood-supplied outer, so-called “red” and central “red-white” zone of the menisci, the healing potential for tears in the inner “white zone” is rather poor and remains a big clinical challenge (Bansal et al., 2021). Although being aware about the poor clinical long-term results (Andersson-Molina et al., 2002; Wachsmuth et al., 2003; Englund and Lohmander, 2004; Mcdermott and Amis, 2006; Intema et al., 2010; Salata et al., 2010), mechanically instable tears at the white zone are still mostly treated by partial meniscectomy, in order to obtain pain relief, avoid tear progression, and allow a faster return to different activities.

Besides biological alterations (Rai et al., 2019), biomechanical changes (Barton et al., 2017; Fischenich et al., 2017b) that are mainly related to the increased tibiofemoral contact pressure (Seitz et al., 2012; Sukopp et al., 2021) have been identified to be responsible for the amplification of PTOA progression after such meniscectomy procedures. Other than the main biomechanical function of tibiofemoral load transmission (Mcdermott et al., 2008), the frequently assigned shock-absorbing function of the menisci might also affect the knee joint homeostasis after meniscectomy. “The shocking truth about meniscus” by Andrews et al. (2011) revealed major flaws of the three most cited publications (Krause et al., 1976; Kurosawa et al., 1980; Voloshin and Wosk, 1983) that affiliate the meniscus with this shock-absorbing ability. Moreover, a recent commentary (Gecelter et al., 2021) came to the conclusion that based both on the mechanical properties and the evolutionary origin, the menisci are not shock absorbers. In contrast, there is evidence assigning a shock-absorbing function directly to the meniscus material itself (Pereira et al., 2014; Gaugler et al., 2015; Coluccino et al., 2017) and to the knee joint during activities of daily living (Winter, 1983; Ratcliffe and Holt, 1997). Therefore, the question remains whether the menisci play an active role as shock absorbers in the healthy, injured, and meniscectomized knee joints or not. Hence, the aim of this biomechanical *in vitro* study was to investigate the shock-absorbing function of the meniscus as an essential integrant of the knee joint during impact and repetitive loads.

Normal menisci have a highly anisotropic structure that is mainly composed of water (75%), collagen (23%), water-binding glycosaminoglycans (1%), and other matrix components leading to a time-dependent viscoelastic behavior (Herwig et al., 1984; Pereira et al., 2014; Seitz et al., 2021). While under static equilibrium conditions (Tibesku et al., 2004; Martin Seitz et al., 2013; Schwer et al., 2020) and repetitive loading (Kessler et al., 2006), the menisci exhibit considerable axial deformation, high impact, or shock loads, as seen, e.g., during jump landings, leading to an increase in their stiffness, and thus, to a decrease in the ability to reduce the transmitted stresses. Therefore, we hypothesize that the menisci contribute significantly to knee joint shock absorption during repetitive loads, while under impact loads, this shock absorption potential is absent.

## 2 Methods

### 2.1 Study Design

The shock-absorbing potential of six porcine knee joints was investigated using a custom developed droptower setup, which provided one degree of freedom. After successful validation of the setup using defined damping materials, the joints were impacted with a total energy of 3.54 J. During the tests, only the meniscus states were successively varied to be able to relate the shock absorption potential directly to the menisci. These were intact, medial meniscus posterior root avulsion, medial meniscectomy, total lateral, and medial meniscectomy. The shock absorption was interpreted by the loss factor, which was calculated *via* the propagation time between two integrated load cells.

After modification of the test setup, a dynamic mechanical analysis (DMA) was conducted on eight porcine knees to investigate their shock-absorbing potential under repetitive, sinusoidal loads. For this purpose, a frequency scan including 10 different frequencies, ranging between 0.1–5 Hz at a constant load amplitude was performed while considering the four different meniscus states. The resultant shock absorption potential is calculated *via* the phase shift between force and displacement (strain) in order to draw conclusions about the influence of the meniscus on the damping in the knee joint.

### 2.2 Specimen Preparation

Fourteen fresh porcine hind limbs (liveweight ≈ 100 kg) were obtained for the experiments from a local butcher. The soft tissues were removed, leaving the capsuloligamentous knee joint structures intact. Before the tibial bone was shortened to a length of 4 cm in distance to the knee joint gap, the head of the fibula was secured using a setscrew. A precision miter screw allowed for plane parallel resection of the femoral bone in 5 cm distance to the joint gap and 45° neutral knee joint flexion (Proffen et al., 2012). During the preparation process, the tissues were kept moist using physiologic saline solution. After the preparation process, the specimens were wrapped in saline saturated gauze and stored at −20°C until the day of testing.

**FIGURE 1:**
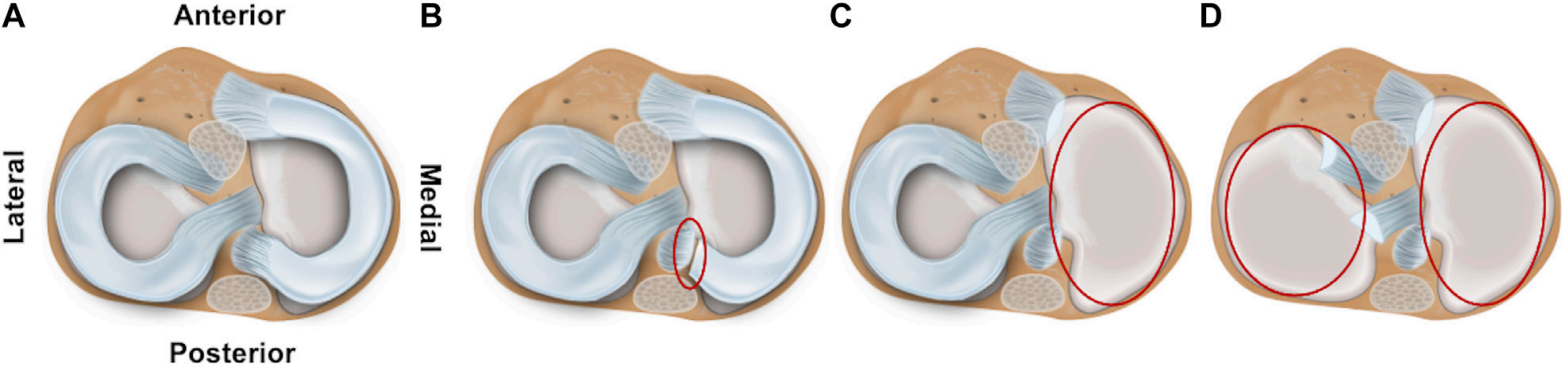
Schematic drawing of the axial view on a tibial plateau of a left knee joint. The femur is removed to allow better visualization of the four investigated meniscal states: **(A)** intact, **(B)** avulsion of the posterior medial meniscus horn, **(C)** medial meniscectomy, and **(D)** total lateral and medial meniscectomy.

Both for the impact and for the repetitive (DMA) loading experiments, four meniscus states (intact, medial posterior root avulsion, medial meniscectomy, and total lateral and medial meniscectomy) were investigated to identify the shock-absorbing function of the meniscus (Figure 1). The meniscus preparation steps were conducted as minimally invasive as possible by an orthopedic surgeon. During these preparation steps, the knee joints were kept in the respective testing setup to avoid a misplacement bias by disassembling and reassembling.

### 2.3 Test Setup and Validation

#### 2.3.1 Droptower Setup

**FIGURE 2:**
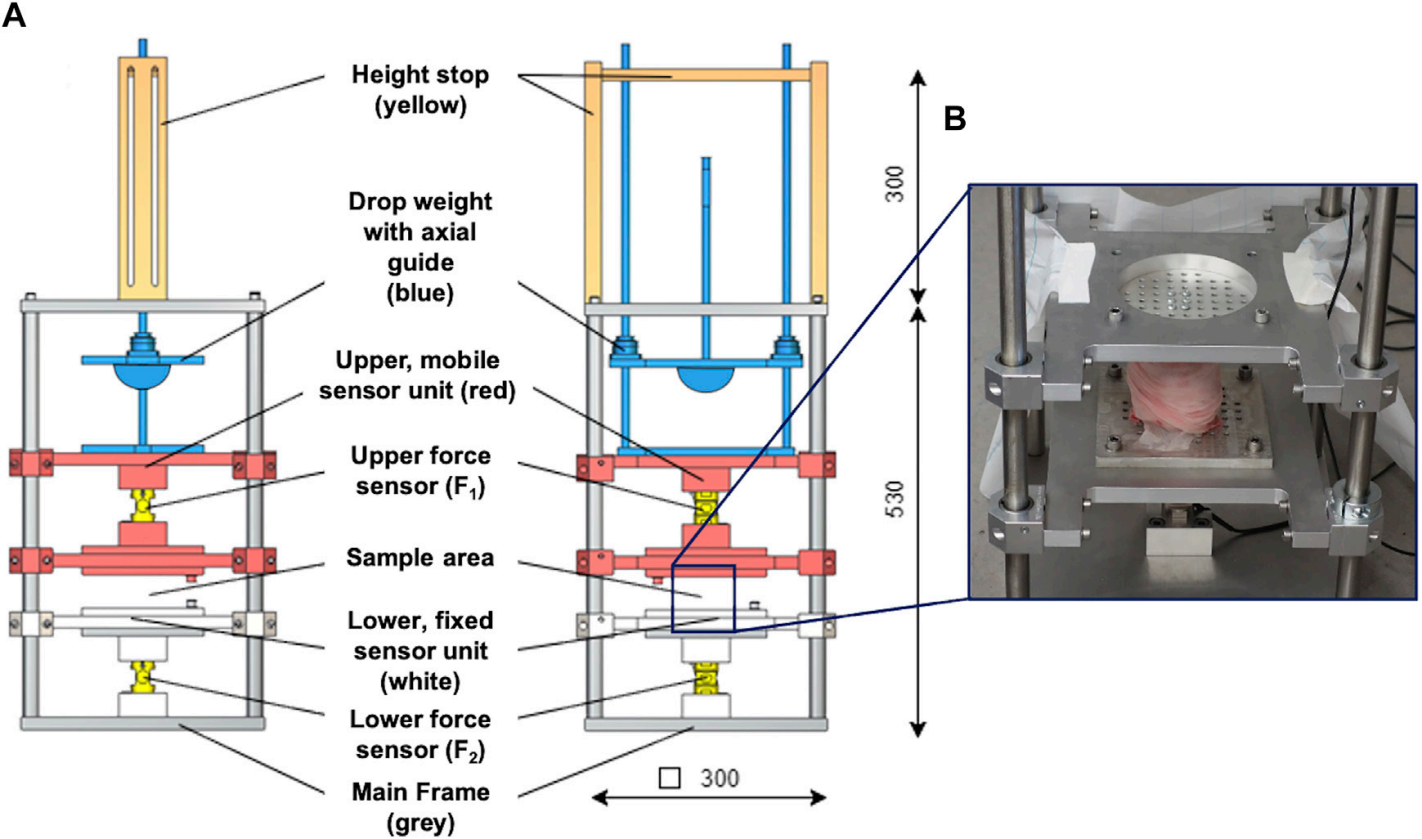
**(A)** Technical drawing of the droptower setup in the side (left) and frontal (middle) view. The setup consisted of a height stop (ochre), which was directly connected to the axial guidance system of the drop weight. A hemisphere impactor (blue) was used to allow for pinpoint impact transmission. The upper, mobile sensor unit (red) is axially guided by eight axial ball bearings and incorporates the upper force sensor. It is further connected to an impact pad at its top end and to a sample holder at the lower end. The lower, fixed sensor unit (white) is also axially guided by four axial ball bearings and incorporates the lower force sensor. The lower end of this sensor unit is directly connected to the main frame. **(B)** Example of an installed porcine knee joint at the sample area at the droptower setup.

The droptower test setup configuration consisted of a weight-adjustable (up to 200 N), axially guided drop weight with a height stop and a hemispherical, stainless-steel impactor, which allows for pinpoint impact transmission (Figure 2). The drop weight hit the impact pad that was mounted to the mobile upper sensor unit containing a screwed-in 10 kN force sensor (KM30z, ME GmbH, Germany) to which the upper sample holder was connected with four knurled screws. Accurate and low-friction axial guidance of the upper sensor unit was ensured using eight linear ball bearings. The test sample was fixed between the two sample holders, while the lower sample holder was axially guided by four linear ball bearings. The lower sensor contained another screwed-in 10 kN force sensor (KM30z, ME GmbH, Germany) that was rigidly connected to the main frame. The test setup provided only one degree of freedom in the axial direction. This was guided by a total of twelve low-friction linear ball bearings, which were in contact with the stainless-steel main frame.

The two series-connected force sensors were used to determine the shock wave propagation by measuring the impact loads at the upper (F_1_) and lower sensor (F_2_) with the corresponding propagation time (Δt). The measurements were used to calculate the loss factor ( η ), which was used to interpret the damping behavior of the test sample. For damping materials, it can be assumed (Jäger et al., 2016) that

η≈2D (1)

while for viscoelastic materials (e.g., articular cartilage or menisci), it can be assumed that

$$\eta =2D\;|\sqrt{1-D^{2}²}|$$ (2)

where *D* is the damping factor and calculated by

$$D=\frac{\text{Λ}}{\sqrt{4\pi^{2}+\text{Λ}{²}^{2}}}$$ (3)

with Λ as the logarithmic decrement, while Λ can be determined by two consecutively measured maxima x_n_:

$${\text{x}}_{\text{n}+1}\text{: }\Lambda =\ln\left(\frac{x_{n}}{x_{n+1}}\right)$$ (4)

**FIGURE 3:**
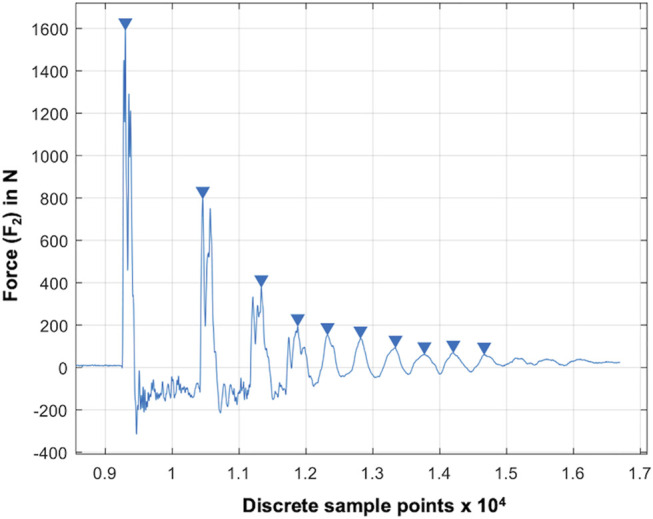
Result graph of a droptower validation test using a known damping material (RF220). The blue arrows indicate automatically determined local maxima (x_n_: blue arrows) at the lower force sensor (F_2_). The force is given over the discrete sample points at a sampling rate of 20 kHz.

We used the maxima at the lower force sensor (F_2_), as they equal to the reaction force, and thus, vary in accordance with the shock absorption ability of the test samples (Figure 3). Based on pretests identifying the potential impact frequencies and following the Nyquist theorem, the sampling rate was set to 30 kHz. Validation of the test setup was conducted using seven special damping materials made of polyurethane foam, indicating specific damping properties and a known loss factor ( η ) ranging from η = 0.11 (PUR RF220, REGUPOL BSW GmbH, Bad Berleburg, Germany) to η = 0.22 (PUR RF740). Each damping material was tested 60 times at two consecutive days by applying an impact of 3.54 J resulting from dropping 2.1 kg at a drop height of 172 mm, which is double of that used by Hoshino et al. (1.77 J) in their experiments (Hoshino and Wallace, 1987). Then, the experimental loss factor was compared to the given loss factor. The resultant Pearson’s correlation coefficient (*r* = 0.93) indicated excellent correlations. Thus, successful validation of the droptower setup configuration was confirmed.

#### 2.3.2 Dynamic Mechanical Analysis Setup

**FIGURE 4:**
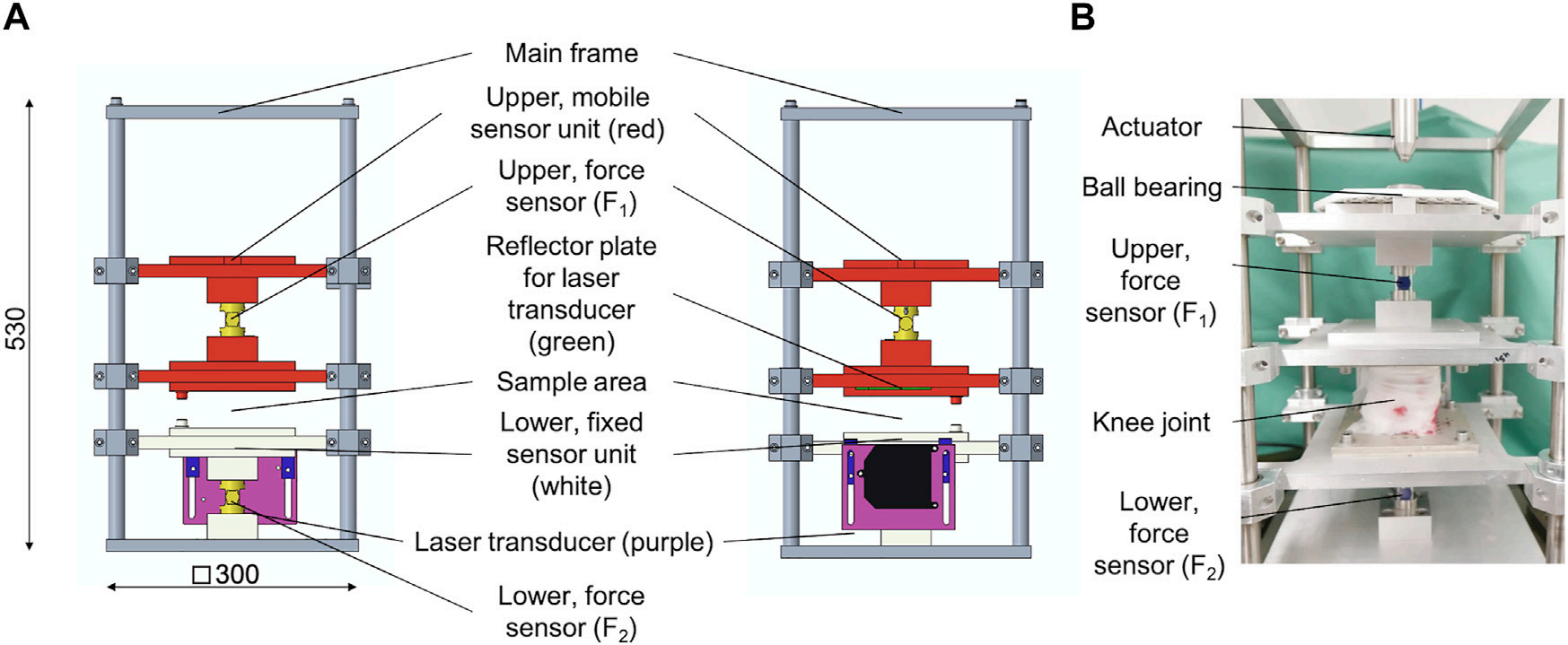
**(A)** Technical drawing of the modified dynamic mechanical analysis (DMA) setup in side (left) and frontal (middle) view. The upper, mobile sensor unit (red) is axially guided by eight axial ball bearings and incorporates the upper force sensor. On its top, there is a multiaxial ball bearing to protect the load cell of the dynamic testing machine, while at the lower end, there is a reflector plate (green) for a laser transducer installed. The lower, fixed sensor unit (white) is axially guided by four axial ball bearings and incorporates the lower force sensor as well as an external, high-precision laser length transducer. The lower end of this sensor unit is directly connected to the main frame. **(B)** Example of an installed porcine knee joint at the sample area at the DMA setup.

**FIGURE 5:**
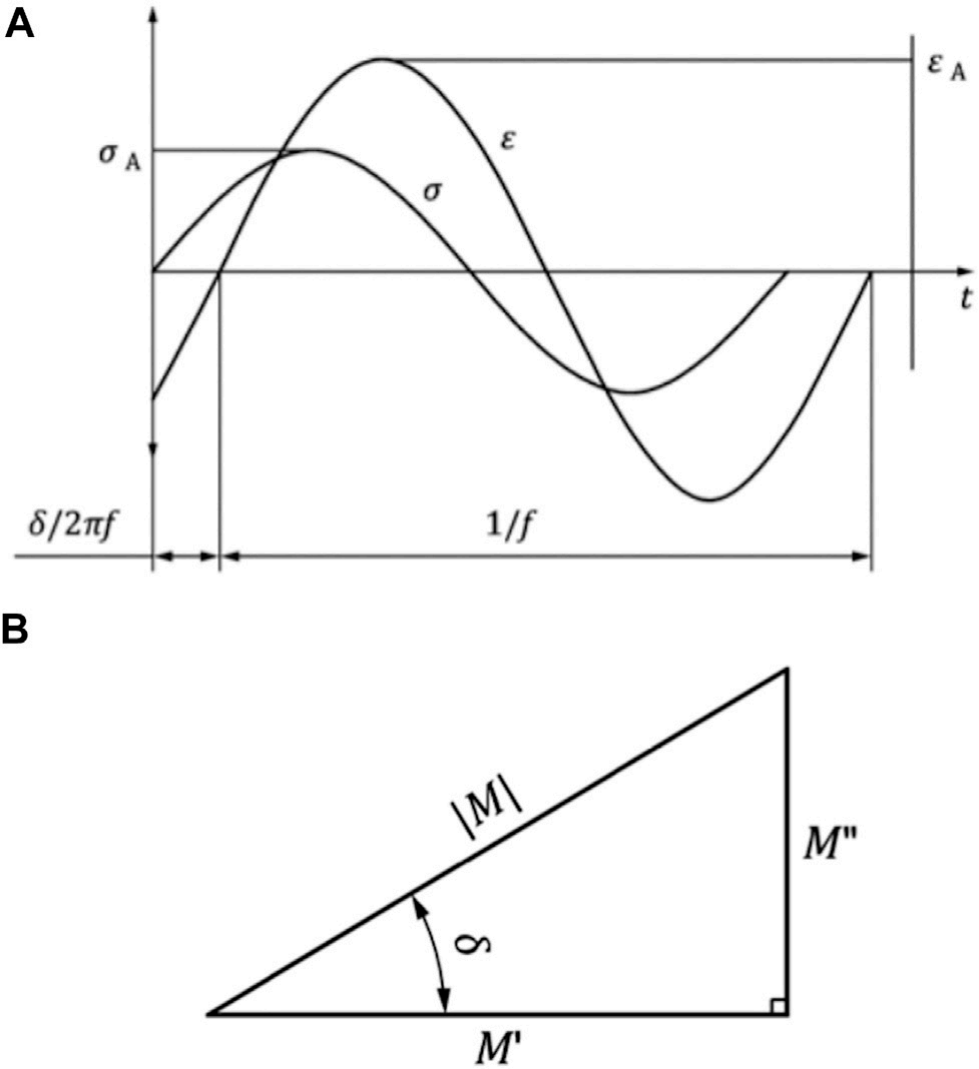
**(A)** Representative force-induced sinusoidal oscillation (F) and the respective deflection response (l) of a viscoelastic material. The time-dependent, characteristic shift (δ/2πf) of the force (F_A_) versus the deflection (l_A_) amplitudes are plotted over the time (t). **(B)** Relation between the storage modulus M′, the loss modulus M″, the phase angle δ, and the magnitude (*M*) of the complex modulus M*. Images are in accordance with DIN EN ISO 6721-1:2018-03.

The modular design of the test setup allows for a fast removal of the height stop and drop weight assembly. This is necessary to allow for an integration of the test setup in the work space of a standard hydraulic dynamic materials testing machine (Instron 8871, Norwood MS, United States) with a total capacity of 10 kN. A multiaxial ball bearing was placed between the upper sensor unit and the actuator of the dynamic materials testing machine (Figure 4) to protect the load cell. The setup was centered and rigidly clamped to the base plate of the materials testing machine. An external high-precision laser length transducer (accuracy: ± 0.6 μm; optoNCDT 2201, Micro-Epsilon Eltrotec GmbH, Göppingen, Germany), which was installed at the lower sensor unit, was used together with its reflection plate counterpart that was firmly connected to the upper sensor unit to measure the sample deflection (l). During the DMA tests, the phase angle (δ), the applied oscillation input (force) amplitude (F_2,A_), and the resultant oscillation output (deflection) amplitude (l_A_) were continuously recorded (Figure 5). Based on these three parameters, the storage modulus (M′) was determined (Ehrenstein et al., 2004) by

$$M^{\prime \;}=\left(\frac{F_{2A}}{l_{A}}\right)cos\delta$$ (5)

while the loss modulus (M″) is calculated by

$$M^{\prime \prime }=\;\left(\frac{F_{2A}}{l_{A}}\right)sin\delta$$ (6)

Based on both moduli, the loss factor (tan δ) can be calculated by

$$tan\;\delta =\;\frac{M\prime \prime }{M\prime }$$ (7)

In addition, the complex modulus E* results from the storage modulus (M′) and the imaginary part (1) of the loss modulus (M″). It is also called dynamic modulus and indicates the behavior of stress to strain under oscillating loads:

$$M^{*}=M\prime +i*M\prime \prime =\;\frac{F_{2A}}{l_{A}}$$ (8)

Validation of the DMA test setup was conducted using the same seven damping materials (PUR RF, REGUPOL BSW GmbH, Bad Berleburg, Germany) as those for the droptower setup validation with a known loss factor ( η=  tanδ). Each damping material was tested three times at 5 Hz sinusoidal loading and a stroke length of ±1.25 mm for 20 cycles. During these validation experiments, the sampling rate was set to 1 kHz. Then, the experimental loss factor was compared to the given loss factor by the manufacturer. The resultant Pearson’s correlation coefficient (*r* = 0.99) indicated excellent correlations. Thus, successful validation of the DMA setup configuration could be confirmed.

Synchronized data acquisition for both the droptower (F_1_, F_2_, Δt) and DMA (F_1_, F_2_, l, and force/displacement output of the materials testing machine) setup measurements was achieved using a USB data acquisition card (USB-6218, NI Corp., Austin TX, United States) and a customized LabVIEW software (LabVIEW 2019; NI Corp., Austin TX, United States). Subsequent data postprocessing was performed using customized MATLAB scripts (MATLAB 2019b; The MathWorks Inc., Natick MA, United States).

### 2.4 Droptower Experiments

Based on the results of a study investigating the impact-absorbing properties of a healthy and meniscectomized knee joint (Hoshino and Wallace, 1987), an *a priori* sample size calculation [G^∗^Power 3.1.9.2 (Faul et al., 2007): *α* = 0.05, Power (1 – *β*) = 0.95, effect size (dz) = 2.79, *n* = 4] was performed to ensure sufficient statistical power [Actual power = 0.98] of the study. In order to be able to identify differences after simulating a meniscus tear, the sample size was increased to *n* = 6.

At the day of testing, the joints were thawed at room temperature and subsequently centrally mounted between the two sample holders using 4–6 proximal and eight distal screws in 45° neutral knee joint flexion (Proffen et al., 2012). To account for the viscoelastic behavior of the knee soft tissues, the joints were loaded 12 min prior to testing with 120 N, following an established protocol (Maher et al., 2017). During preconditioning, the drop height was adjusted to 172 mm and the drop weight was adjusted to 2.1 kg, resulting in an impact energy of 3.54 J, which is double of that used by Hoshino et al. (1.77 J) (Hoshino and Wallace, 1987). After application of the impact and successful crosscheck of the data recording, the joint was unloaded and allowed to undergo relaxation for 12 min. The tests were repeated three times, resulting in a total test time of 90 min for each meniscus state. The mean values of these three measurements were used for further analyses. Then, the next meniscus preparation step was performed and the experiments were repeated. After each preparation step the joint capsule incision was fixed by surgical sutures. The sampling rate during the droptower experiments was set to 30 kHz. During the tests, the joints were kept moist by constantly misting physiological saline solution.

### 2.5 Dynamic Mechanical Analysis

Based on the results of a similar study performing a DMA on human menisci (Pereira et al., 2014), an *a priori* sample size calculation [G∗Power 3.1.9.2 (Faul et al., 2007): *α* = 0.05, Power (1−β) = 0.80, effect size (dz) = 1.15, *n* = 7] was performed to ensure sufficient statistical power [Actual power = 0.85] of the study. Comparators to determine the effect size dz were tan δ values (mean ± SD) of the anterior medial menisci and mid body medial menisci values. In order to be able to identify differences after simulating meniscus pathologies, the sample size was increased to *n* = 8.

At the day of testing, the porcine knee joints were thawed and centrally applied in the modified DMA test setup in 45° neutral knee joint flexion (Proffen et al., 2012). A total of 8–12 screws were used to secure the specimen against translation. Taylor et al. (2006) identified an axial knee joint loading of 2.12 times body weight (BW) during the stance phase and 0.3 times BW during the swing phase of normal gait in sheep knees. Since sheep and pigs are similar in their neutral knee position and gait (Proffen et al., 2012), these values were transferred to the forces in the porcine knee, resulting in a static load of 1,210 N and a superimposed sinusoidal load of 910N, which was applied to the porcine knee joints. As a result, peak loads of 2120 N and minimum loads of 300 N were identified for the sinusoidal DMA tests. Additionally, the joints were loaded 12 min prior to testing with 300 N to account for the viscoelastic behavior of the knee soft tissues (Maher et al., 2017). A total of ten frequencies (0.1, 0.2, 0.4, 0.6, 0.8, 1, 2, 3, 4, and 5 Hz) were arranged randomly for each knee and scanned directly one after the other, in accordance with this randomized sequence. The frequency range was adapted from Pereira et al. (2014) and cropped to the frequencies that match best to those observed during gait, running, and other daily activities (Danion et al., 2003; Simoni et al., 2021). The sampling rate was adapted to the respective frequency in a way that 1,000 values were recorded per single sinusoidal oscillation, resulting in a total of 10,000 measurement points per DMA run. After the intact meniscus state investigation, the joints were allowed to undergo relaxation for 12 min. Then, the next meniscus preparation step was performed and the experiments were repeated. After each preparation step, the joint capsule incision was fixed by surgical sutures. During the tests, the joints were continuously kept moist by constantly misting physiological saline solution.

### 2.6 Statistical Analysis

Gaussian distribution of the results data was tested using the Shapiro–Wilk test, resulting in non-normally distributed data for the droptower and normally distributed data for the DMA experiments. Thus, non-parametric (droptower) and parametric (DMA) statistical analyses were performed using a statistical software package (SPSS v24, IBM Corp., Armonk, NY, United States).

In detail, for the droptower experiments, F_2_ and η were elaborated by comparing the four meniscus states using a Friedman test followed by a Wilcoxon test, in which significant differences were observed. For the DMA experiments, tan δ was elaborated by comparing the four meniscus states by means of a one-way ANOVA followed by Duncan post-hoc testing, in which significant differences were observed. In general, *p* = 0.05 was considered significant, while *p*-value Bonferroni correction was applied where necessary.

## 3 Results

### 3.1 Droptower Experiments

**FIGURE 6:**
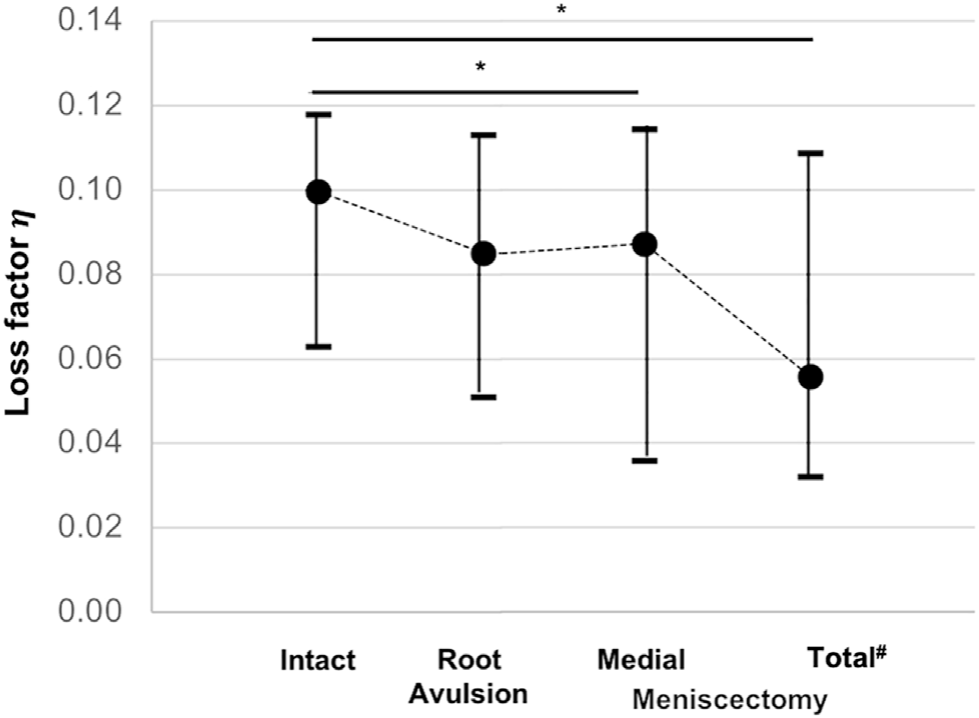
Minimum, median, and maximum loss factor ( η ) values compared at the four investigated meniscus states: intact, root avulsion at the posterior horn of the medial meniscus, medial meniscectomy and total lateral and medial meniscectomy. The dotted line indicates the progression of the consecutively investigated meniscus states. ^#^The total meniscectomy state was statistically lower than any of the other states; *Wilcoxon test: ^*,#^ *p* < 0.05; *n* = 6.

Friedman testing revealed significant differences in both the force maxima at the lower force sensor (F_2_; *p* = 0.02) and the loss factor ( η ; *p* = 0.002). For the intact state, a median F_2_ of 1187 N was measured. Furthermore, a significant F_2_ increase of 9% after medial meniscectomy (Wilcoxon: *p* = 0.46) and of 24% after total meniscectomy (Wilcoxon: *p* = 0.014) was identified. These findings were completed by the results of the loss factor (Figure 6): The intact state possessed a medial loss factor of η = 0.1. Friedman testing revealed significant differences of the loss factor ( η ) for the meniscus states (*p* = 0.002). In detail, except for the root avulsion state (−15%, Wilcoxon: *p* = 0.12), the loss factor significantly decreased (Wilcoxon: *p* < 0.046) by a maximum of −68% for the total meniscectomy state (Wilcoxon: *p* = 0.028) when compared to the intact state. The loss factor of the total meniscectomy state was always statistically lower (Wilcoxon *p* = 0.028) than those of any other state. However, there was no loss factor difference for the comparison between the posterior root avulsion and the medial meniscectomy state (Wilcoxon *p* = 0.054).

### 3.2 Dynamic Mechanical Analysis

**FIGURE 7:**
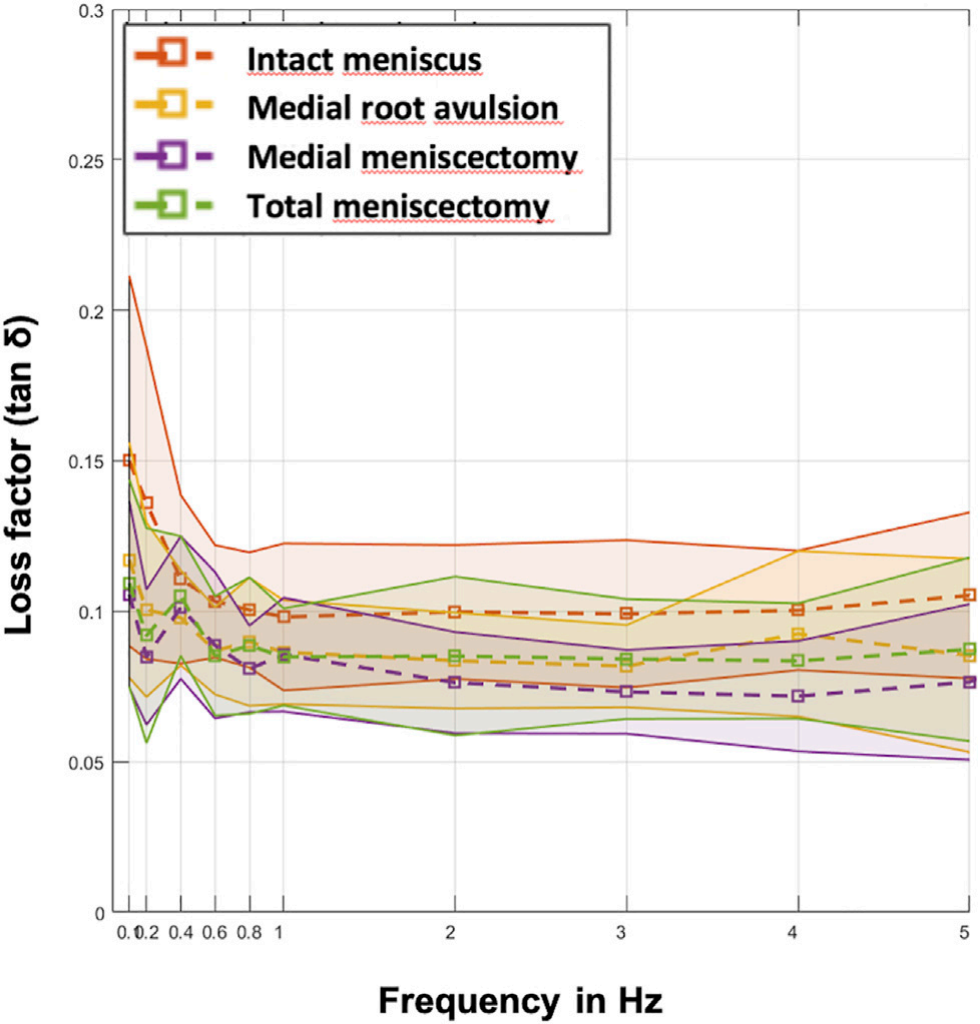
Mean values (dashed lines) of the loss factor (tan δ) ± SD (represented by the envelope in the same color) plotted over the ten investigated frequencies (0.1, 0.2, 0.4, 0.6, 0.8, 1, 2, 3, 4, and 5 Hz) at the four meniscal states (red: intact menisci; yellow: avulsion of the posterior medial meniscus horn; purple: medial meniscectomy; green: total lateral and medial meniscectomy). *n* = 8.

**FIGURE 8:**
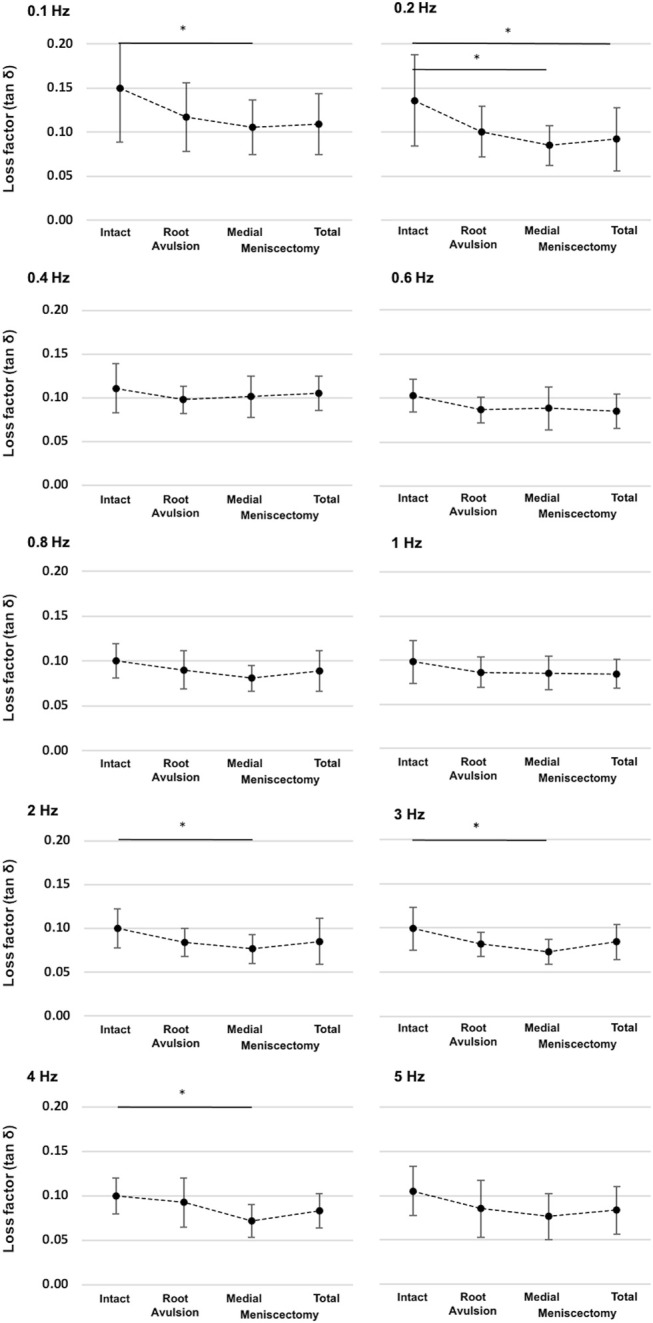
Detailed representation of the loss factor (tan δ) ± SD at the ten investigated frequencies (0.1–5 Hz) and under the four investigated meniscus states: intact, root avulsion at the posterior horn of the medial meniscus, medial meniscectomy, and total lateral and medial meniscectomy. The dotted line indicates the progression of the consecutively investigated meniscus states. *One-way ANOVA with post-hoc Duncan test: *p < 0.05; *n* = 8.

Parametric statistical analyses revealed that knees with an intact meniscus had the highest loss factors, ranging from tan δ = 0.10 to tan δ = 0.15 throughout all investigated frequencies (Figure 7). Each consecutive simulated meniscus deterioration lowered the loss factor. Thus, after posterior medial root avulsion, the mean value of the loss factor (tan δ) was reduced by 15%, while medial meniscectomy resulted in a reduction of 24%. The resection of both the lateral and medial menisci resulted in a reduction of 18% compared to the intact meniscus state. When comparing the meniscal states at specific frequencies in detail (Figure 8), one-way ANOVA with Duncan post-hoc testing indicated significant differences for the comparisons of the intact state and the medial meniscectomy at 0.1, 0.2, 2, 3, and 4 Hz (*p* < 0.04) and for the comparisons of the intact meniscus state and the total meniscectomy at 0.2 Hz (*p* = 0.02).

## 4 Discussion

In this biomechanical *in vitro* study, we investigated the shock-absorbing potential of the menisci inside porcine knee joints during impact loading and repetitive sinusoidal loads by applying a DMA. The test setup was successfully validated for both applications. Both during the impact loads and during the DMA analysis, the loss factor ( η ) and the loss factor (tan δ), respectively, were the highest for the intact meniscus state. During impact loading, the loss factor and, thus, the shock-absorbing potential of the menisci were reduced the more meniscus tissue was resected. Therefore, this study suggests that the menisci contribute to the shock absorption inside porcine knee joints under impact loads. During repetitive sinusoidal loads, the loss factor was lowest for the medial meniscectomy state, followed by the total lateral and medial meniscectomy state. However, both meniscectomy states indicated significantly lower loss factors compared to the intact meniscus state. Hence, it can be concluded that under sinusoidal loads in a frequency range from 0.1 Hz up to 5 Hz, the menisci significantly contribute to the knee joint shock absorption. In summary, the results of the present study suggest that the menisci contribute to the shock absorption during both repetitive and impact loading, refuting our hypothesis.

Hoshino and Wallace (1987) investigated the shock absorption potential of human menisci in 20 knee joints and found a comparable reaction force increase of 21% for the comparison between the intact and the total meniscectomized knee joint. Also, for the root avulsion and medial meniscectomy conditions, our relative values were in a very similar range (108–109%). Regarding the force at the lower sensor (F_2_), Hoshino et al. determined forces of up to 1600 N, thus being 400 N higher compared to the F_2_ forces of the present study. This might be due to the more pronounced curvature of the porcine tibial, potentially resulting in higher shear forces, which might be transmitted to other joint structures in the porcine knee. Chu et al. (1986) investigated the shock wave propagation during dynamic loading in six human lower limbs using accelerometers and force transducers. They measured an average increase in impulse force of 11% at the tibia and 23% at the femur, after total meniscectomy and removal of the articular cartilage. In the present study, F_2_ increased by 24% only after total meniscectomy. In contrast, Chu et al. also removed cartilage structures. Articular cartilage has been shown to possess a 40-times higher complex modulus (E*) (Temple et al., 2016) compared to the menisci (Pereira et al., 2014; Fischenich et al., 2017a). This might be interpreted in a way that the menisci seem to be more responsible for the increased tibial forces in the Chu study (Chu et al., 1986). Therefore, the results of Chu et al. differ by only 1% to the results of the present study. In their work, Kurosawa et al. (1980) examined a total of 14 human knee joints under quasi-static loads of 500, 1,000, and 1,500 N at a loading rate of 5 mm/min. They observed higher energy dissipation in meniscectomized knee joints than intact knee joints at all loading levels. These findings are in contrast to the ones obtained in the present study and to those from Hoshino and Wallace (1987) and Chu et al. (1986). However, compared to the other studies, the transmitted energy during the loading reached only 0.023 J at a load of 1500 N. Thus, the results from Kurosawa are difficult to compare with the present study and are more likely to reflect the energy absorption potential during creep loading of all viscoelastic knee joint structures.

Pereira et al. (2014) investigated in their study the viscoelastic response of isolated human meniscus tissue at a frequency range of 0.1–10 Hz under displacement-controlled conditions. They subdivided the menisci into their anatomical regions (anterior horn, pars intermedia, posterior horn) and determined variations of the loss factor (tan δ) at 1 Hz from 0.12 at the posterior lateral meniscus to 0.18 in the anterior medial meniscus. In accordance to the findings of the present study, Pereira also showed a frequency dependent response: while at low frequencies (0.1–0.6 Hz) high loss factor values (∼0.2) and a loss factor minimum at 1 Hz were observed, high frequencies (>3 Hz) again led to high loss factor values. Gaugler et al. (2015) investigated in their study on isolated meniscus tissue both sinusoidal loads at a frequency of 0.1 Hz and impact loads and found loss factors of 0.3 for sinusoidal and 0.17 for impact loads. The findings of our study are within the same range as those from Gaugler et al. and indicate the same ratio when comparing sinusoidal and impact loads. Additionally, Coluccino et al. (2017) investigated the viscoelastic response of bovine meniscus explants and found both similar ranges of the loss factor (tan δ) of, e.g., 0.17 at 0.1 Hz and a similar frequency dependency of the damping response of the meniscus within the range from 0.1 to 5 Hz.

Limitations should be considered when interpreting the results of the present *in vitro* study. First, due to the very good availability and standardized phenotype, we used a porcine knee joint model in our study investigation. Although the porcine knee showed only acceptable appearance congruence for the ACL and lateral menisci (Proffen et al., 2012) and differences in the viscoelastic properties (Sandmann et al., 2013) when compared to the human knee joint, the relative comparisons were in a similar range than those observed in a human cadaver study, both for the impact (Hoshino and Wallace, 1987) and the DMA conditions investigating isolated menisci [tan δ ∼ 0.2; (Pereira et al., 2014)]. Furthermore, the test setup provided only one translational degree of freedom in the axial direction. However, during pretests, we experienced significant evasive rotational movement of the porcine knee joint under compressive loads, especially after the two meniscectomy states. In consequence, the obtained shock absorption results were significantly biased by these evasive movements and the resultant influence of the viscoelastic response of the surrounding soft tissues, while providing more physiological degrees of freedom.

## 5 Conclusion and Outlook

The results of this biomechanical *in vitro* study indicate that the meniscus significantly contributes to the shock absorption of the porcine knee joint both under impact loads and under more moderate, sinusoidal loads at different frequencies. The findings may have an impact on the rehabilitation of young, meniscectomized patients that want to return to sports as soon as possible. Such patients are exposed to critical loads carried by the articular cartilage, when performing shock intensive sports like skiing, volleyball, etc. In summary, a partial meniscectomy leads not only to a reduced tibiofemoral contact area, but also to an inhibited shock-absorbing potential of the meniscus and, thus, to an increased contact pressure. Consequently, during the meniscectomy procedure, care should be taken to save as much meniscal tissue as possible, underlining the clinical findings to save the meniscus (Lubowitz and Poehling, 2011; Seil and Becker, 2016; Lee et al., 2019; Pujol and Beaufils, 2019)—also from a shock absorption point of view. A meniscus allograft transplantation (MAT) procedure is able to biomechanically restore the injured or (partially) meniscectomized knee joint to the original, healthy state (Koh et al., 2019; Seitz and Durselen, 2019; Zaffagnini et al., 2019). However, not only from a biomechanical point of view, but also from a clinical point of view, especially when patients present persisting post-meniscectomy syndromes, does MAT represent a viable option to decrease pain, improve knee joint function, and, thus, delay the onset of PTOA after meniscus injury (Zaffagnini et al., 2016; De Bruycker et al., 2017).

## Data Availability

The raw data supporting the conclusion of this article will be made available by the authors, without undue reservation.
